# Exosome-Based Proteomic Profiling for Biomarker Discovery in Pediatric Fabry Disease: Insights into Early Diagnosis Monitoring

**DOI:** 10.3390/biomedicines13112598

**Published:** 2025-10-23

**Authors:** Zhihong Lu, Yu Xia, Bingying Wang, Pingping Jiang, Jianhua Mao

**Affiliations:** 1 Department of Nephrology, National Clinical Research Center for Child Health, Children’s Hospital, Zhejiang University School of Medicine, Hangzhou 310052, China; lzhsjf@zju.edu.cn (Z.L.); 12218528@zju.edu.cn (Y.X.); 2 School of Basic Medical Sciences of Zhejiang University, Hangzhou 310052, China; 22218003@zju.edu.cn; 3 Institute of Pharmaceutical Biotechnology, Zhejiang University School of Medicine, Hangzhou 310058, China

**Keywords:** Fabry disease, child, exosome, biomarkers

## Abstract

**Background**: Fabry disease (FD) is an X-linked lysosomal storage disorder caused by *GLA* mutations, leading to deficient α-galactosidase A (α-Gal A) activity and progressive glycosphingolipid accumulation. While α-Gal A activity is the diagnostic gold standard, its sensitivity is reduced in late-onset or heterozygous patients. Conventional biomarkers such as lyso-Gb3 provide only limited insight into disease progression and therapeutic response. Exosomes, as stable carriers of disease-specific proteins, may offer complementary biomarkers for early detection and longitudinal monitoring. **Methods**: Twenty-one pediatric FD patients with confirmed GLA mutations were enrolled. Clinical, enzymatic, renal, and cardiac parameters were assessed. Plasma-derived exosomes were characterized by transmission electron microscopy and proteomic profiling. Differentially expressed proteins were identified using mass spectrometry, analyzed using GO/KEGG enrichment, and validated using RT-PCR, ELISA, and immunofluorescence in patient samples and *Gla*^−/−^ mice. **Results**: Male patients showed markedly reduced α-Gal A activity and elevated lyso-Gb3 compared with females. Although overt renal and cardiac dysfunction was uncommon, several patients exhibited early abnormalities such as proteinuria, an elevated LVMI, or increased cTnI levels. Proteomic analysis identified 2553 proteins, of which 188 were differentially expressed. Fibrosis- and inflammation-related proteins, including THBS1 and CFHR5, were upregulated, while protective factors such as APM1, SERPINA10, and CAB39 were downregulated. IGFBP3 was also elevated and closely linked to tissue remodeling. Enriched pathways were involved in PPAR/AMPK signaling, lipid metabolism, and complement activation. **Conclusions**: Exosomal proteomic profiling revealed early molecular signatures of cardiorenal involvement in pediatric FD. Key proteins such as THBS1, CFHR5, IGFBP3, APM1, and CAB39 show strong potential as biomarkers for risk stratification, disease monitoring, and therapeutic evaluation.

## 1. Introduction

Fabry disease (FD, OMIM 301500) is an inherited X-linked lysosomal storage disorder caused by pathogenic variants in the *GLA* gene, leading to deficient activity of the lysosomal enzyme α-galactosidase A (α-Gal A). This enzymatic defect results in systemic accumulation of globotriaosylceramide (Gb3) and related glycosphingolipids across multiple tissues. Pediatric patients manifest as a progressive multiorgan involvement that primarily affects the kidneys, heart, and nervous system, with these organ complications accounting for most of the disease burden, morbidity, and mortality [1,2].

The estimated incidence in males ranges from 1:40,000 to 1:117,000. However, newborn screening studies have revealed a greater prevalence of late-onset variants, suggesting that FD remains substantially underdiagnosed in the general population. Early symptoms, such as acroparesthesias, angiokeratomas, hypohidrosis, and corneal verticillata, often arise during childhood but are nonspecific, frequently delaying accurate diagnosis [2].

With the availability of enzyme replacement therapy (ERT) and pharmacological chaperones, early identification and timely intervention have become critical to prevent irreversible organ damage [3,4]. Accordingly, the identification of sensitive and dynamic biomarkers is essential to improve screening accuracy, diagnosis, and disease monitoring in FD [5]. Plasma globotriaosylsphingosine (Lyso-Gb3) is recognized as a robust biomarker, yet its ability to fully reflect disease heterogeneity and therapeutic response remains limited. Biomarkers play a crucial role in the screening and diagnosis of FD [6].

Extracellular vesicles, particularly exosomes, have recently emerged as promising sources of disease biomarkers. Exosomes are nanosized vesicles (30–150 nm) secreted by nearly all cell types, encapsulated by a lipid bilayer, and enriched in proteins, lipids, and nucleic acids that are actively involved in intercellular communication [7]. Compared with soluble plasma biomarkers, exosomal cargos are less affected by abundant serum proteins and exhibit enhanced stability, thereby offering unique advantages for clinical applications [8]. Proteomic analyses of exosomes have already provided valuable insights into several metabolic and lysosomal storage disorders [9]. However, to date, no systematic studies have investigated the proteomic profile of plasma exosomes in Fabry disease [10]. This knowledge gap highlights an important and unexplored opportunity for discovering novel, clinically relevant biomarkers that may improve disease stratification and monitoring [11].

Therefore, this study aimed to perform a comprehensive proteomic characterization of plasma-derived exosomes from patients with FD. By systematically profiling the protein cargo of exosomes, we sought to identify novel biomarker candidates that may complement or surpass existing markers such as Lyso-Gb3. Given the rarity of pediatric FD and the limited availability of treatment-naïve samples, we designed this as a pilot exploratory study to generate preliminary molecular insights into exosome-based biomarkers.

## 2. Materials and Methods

### 2.1. Patient and Specimen Information

Clinical data of 21 children with FD diagnosed at the Children’s Hospital Affiliated with Zhejiang University School of Medicine between July 2014 and August 2023 were collected. Inclusion criteria: Children with confirmed diagnosis of FD. The diagnosis was made by qualified clinicians at the Children’s Hospital Affiliated to Zhejiang University School of Medicine. Diagnostic criteria were based on the Expert Consensus on Diagnosis and Treatment of FD in China (2021 Edition): male children with clinical compliance, reduced α-Gal A enzyme activity, and carrying pathogenic mutations of the *GLA* gene; or female children carrying pathogenic mutations of the *GLA* gene. To ensure that the exosomal proteomic findings reflected the untreated disease state, only patients who had not received ERT or any other specific treatment before blood sample collection were enrolled in this study.

Data collection: (1) Age: Age of onset and age at diagnosis. (2) Gender. (3) Symptoms and signs: Presence of pre-existing symptoms such as paresthesia, pain, cutaneous angiosarcoma, hypohidrosis or anhidrosis, gastrointestinal discomfort, visual/hearing disturbances, proteinuria, and blood pressure. (4) *GLA* gene mutation site and family history of the disorder.

For the exosomal protein analysis, we included three age- and sex-matched male FD patients and three healthy male controls. Plasma samples (1 mL) were collected from both the FD children and the age- and sex-matched healthy controls, followed by centrifugation at 1000× *g* for 10 min. The supernatant was carefully collected and aliquoted into sterile EP tubes, with each aliquot containing 500 μL, and stored at −80 °C for subsequent analysis.

### 2.2. Exosome Extraction and Detection

Exosomes were extracted from plasma using the Hieff^®^ Quick Exosome Isolation Kit (Yeasen, Shanghai, China). First, 1 mL of plasma was thawed on ice and centrifuged at 4 °C to remove debris. Four times the plasma volume of sterile phosphate-buffered saline (PBS) was added and mixed thoroughly. The recommended amount of exosome isolation reagent was then added, and the mixture was incubated overnight at 4 °C. Following incubation, the mixture was centrifuged at 10,000× *g* for 60 min at 4 °C to precipitate exosomes, which were subsequently resuspended in PBS for further analysis.

We used TSG101 (Proteintech, Wuhan, China; 28283-1-AP 1:7500) as a positive exosomal marker and Calnexin (Proteintech, Wuhan, China; 10427-2-AP 1:20,000) as a negative control marker by Western blot.

To visualize the exosomes, the resuspended samples were fixed in 4% paraformaldehyde (PFA) and imaged using a JEM-1230 transmission electron microscope (TEM) (JEOL, Tokyo, Japan), with images captured by an Olympus EM208S TEM (Hitachi, Tokyo, Japan).

### 2.3. Exosomal Protein Analysis via Mass Spectrometry

Two mobile phases were prepared for mass spectrometry analysis: Phase A consisted of 100% water with 0.1% formic acid, and Phase B comprised 100% acetonitrile with 0.1% formic acid. Samples were reconstituted in 10 μL of Phase A and centrifuged at 14,000× *g* for 20 min at 4 °C. The supernatant was collected for liquid chromatography–mass spectrometry (LC-MS) analysis.

The analysis utilized a nanoElute ultra-high-performance liquid chromatography (UHPLC) system coupled with a timsTOF Pro2 mass spectrometer (Agilent Technologies Inc., Santa Clara, CA, USA). The mass spectrometry parameters included an ion spray voltage of 1.5 kV and a mass-to-charge (*m*/*z*) scan range of 100–1700. The PASEF mode was employed for ten MS/MS scans, with a total cycle time of 1.17 s.

Mass spectrometry data were exported to Excel, and peptide spectrum matching (PSM) was used to assess protein expression. PSM values were normalized and multiplied by 100 to obtain PSM%. Statistical analysis, using a two-tailed independent *t*-test, identified significantly differentially expressed proteins between the FD and control (CON) groups (*p* < 0.05).

### 2.4. Cell Culture

The *Gla*^−/−^ HEK293 cell line was constructed using a Cas9 and sgRNA (TTGGAAATAAAACCTGCGC) expression vector. Target cells were infected via transient transfection, enabling genomic editing, followed by clonal selection, expansion, and Sanger sequencing validation (Figure S2).

For cell culture, DMEM was chosen as the growth medium, supplemented with fetal bovine serum and appropriate antibiotics, such as penicillin and streptomycin, to prevent contamination. Cells were incubated in a 37 °C humidified incubator with a 5% CO_2_ atmosphere. Upon reaching confluency, cells were passaged, and the medium was replaced every 2–3 days.

### 2.5. Immunofluorescence (IF)

Immunofluorescence staining was performed on renal tissues from *Gla*^−/−^ mice (B6;129-Glatm1Kul/J, Stock No. 003535, the Jackson Laboratory, Bar Harbor, ME, USA). Paraffin-embedded sections were deparaffinized, rehydrated, and subjected to heat-induced antigen retrieval. After circumscribing to prevent evaporation, sections were blocked with 5% bovine serum albumin (BSA) and incubated overnight at 4 °C with primary antibodies against CAB39 (1:200, ab51132, Abcam, Cambridge, UK), thrombospondin-1 (THBS1, 1:200, ab267388, Abcam, UK), and insulin-like growth factor binding protein-3 (IGFBP3, 1:200, ab220429, Abcam, UK). Following PBS washes, sections were incubated with species-appropriate fluorophore-conjugated secondary antibodies, and nuclei were counterstained with DAPI. Images were acquired using a confocal laser scanning microscope (FV3000, Olympus, Tokyo, Japan). For each experiment, five mice (three males and two females) were used, and fluorescence intensity was quantified in three randomly selected fields per mouse.

### 2.6. Enzyme-Linked Immunosorbent Assay (ELISA)

For the ELISA, the levels of THBS1, IGFBP3, and APM1 in the plasma of FD patients and healthy children were measured using kits from Xiamen Lun Chang Shuo Biotechnology Co., Ltd. (Wuhan, China), following the manufacturer’s instructions. Briefly, samples and HRP-conjugated detection antibodies were added to the wells, followed by incubation and washing. The substrate, 3,3′,5,5′-tetramethylbenzidine (TMB), was then added and incubated. The absorbance at 450 nm was measured using a microplate reader (Thermo Multiskan FC, Waltham, MA, USA).

### 2.7. Quantitative Reverse Transcription PCR (qPCR)

Total RNA was extracted using the SteadyPure RNA Extraction Kit (Accurate Biotechnology, Changsha, Hunan, China) and reverse-transcribed into cDNA with the Evo M-MLV RT Mix Kit (Accurate Biotechnology, Changsha, Hunan, China). Real-time quantitative PCR was conducted using the SYBR Green Pro Taq HS qPCR Kit (Accurate Biotechnology, Changsha, Hunan, China) to assess the relative mRNA levels of *Β-ACTIN* and apolipoprotein genes (*APOA2*, *APOC1*, *APOC2*, *APOL1*), *FGA*, *SERPIND1*, *SERPINF2*, SERPINA10, *THBS1*, *IGFBP3*, *A1BG*, *AGT*, *CFHR5*, and *AMBP*. The thermal cycling conditions were as follows: 95 °C for 30 s, followed by 40 cycles at 95 °C for 5 s and 60 °C for 30 s. The primers used are listed in Supplementary Table S1.

### 2.8. Bioinformatics Analysis

Gene Ontology (GO) and Kyoto Encyclopedia of Genes and Genomes (KEGG) enrichment analyses were performed using the clusterProfiler package (v4.6.2) in R. GO annotations were obtained via InterProScan (5.52-86.0), incorporating Pfam, PRINTS, ProDom, SMART, ProSite, and PANTHER databases. Enrichment was based on a hypergeometric test with Benjamini–Hochberg FDR correction. Pathways with FDR < 0.05 and gene count ≥3 were considered significant, using all identified proteins as the background.

Differentially expressed proteins (DEPs) were defined by fold change ≥1.5 and FDR < 0.05. Results were visualized with volcano plots and hierarchical clustering heatmaps. Protein–protein interaction (PPI) networks were predicted using STRING (v11.5) with a confidence score ≥0.7 and visualized via Cytoscape (v3.9.1).

### 2.9. Statistical Analysis

All experiment were conducted independently at least three times. Data are presented as means ± standard deviation (SD). Statistical analyses were performed using SPSS 22.0. Levene’s test was used to assess homogeneity of variance, and when variance was homogeneous, independent samples *t*-tests were employed. A *p*-value of <0.05 was considered statistically significant.

## 3. Results

### 3.1. Patient Information

This study enrolled 21 pediatric patients with FD; the median age at diagnosis was 10 years (ranging from 6 to 17 years) and the majority were male (n = 16), consistent with the X-linked inheritance pattern. In total, 12 patients (57.1%) were diagnosed via family screening following identification of a proband, whereas 9 patients (42.9%) were detected using high-risk screening prompted by symptoms such as neuropathic pain or hypohidrosis. The diagnostic delay was considerable, with the interval from symptom onset to confirmed diagnosis ranging from 8 months to 7 years (mean: 2.4 ± 2.1 years).

All patients carried pathogenic or likely pathogenic variants in the *GLA* gene, with recurrent mutations including R112H (c.335G>A), N34H (c.100A>G), and G261V (c.782G>T). Most variants were maternally inherited, with one de novo case identified. Enzyme assays demonstrated markedly reduced α-Gal A activity in all male patients, with values as low as 0.26 μmol/L/h in plasma. Female patients exhibited variable levels of residual enzyme activity, ranging from near-normal to significantly reduced activity, consistent with lyonization effects. Lyso-GL-3 levels, a sensitive biomarker of disease activity, were elevated in nearly all patients (range: 0.51–115.63 ng/mL). Several male patients showed extremely high levels (>70 ng/mL), reflecting significant glycosphingolipid accumulation.

Clinically, most symptomatic patients exhibited classic early-onset manifestations of FD, especially males. Acroparesthesia and hypohidrosis or anhidrosis were the most frequently reported symptoms, while some patients also had gastrointestinal complaints or angiokeratomas. In contrast, many female patients were either asymptomatic or displayed milder manifestations, reflecting the variable penetrance and expression in heterozygous individuals. These findings underscore the phenotypic diversity of FD in pediatric patients and highlight the importance of combining enzymatic, genetic, and clinical screening approaches to enable timely diagnosis and intervention (Table 1). 

### 3.2. Renal and Cardiac Function in Fabry Patients

Renal and cardiac assessments were performed in all 21 patients to evaluate organ involvement. Serum creatinine levels ranged from 30 to 85 μmol/L, generally within age-appropriate reference ranges. The estimated glomerular filtration rate (eGFR) showed wider variability (74.7–176.4 mL/min/1.73 m^2^), with the lowest value observed in patient 1, suggesting early renal impairment. Microalbuminuria, assessed by the urine albumin-to-creatinine ratio, ranged from 2.2 to 30.3 mg/g·Cr. The highest level occurred in patient 10, consistent with glomerular involvement. Microscopic hematuria was present in 13 patients, though most had low red blood cell counts (<6 cells/HPF), except for 1 patient with markedly elevated levels (>200 cells/HPF). Twenty-four-hour urinary protein excretion levels ranged from 36.2 to 209 mg, with mild elevations in several cases, indicating early glomerular dysfunction despite preserved eGFR.

Cardiac troponin I (cTnI) levels were within normal limits in most patients (0.01–0.19 ng/mL). Elevated levels were noted in patients 11, 18, and 21, potentially reflecting subclinical myocardial involvement. The left-ventricular mass index (LVMI) ranged from 23.6 to 52.3 g/m^2.7^. While values were largely within normal limits, several patients—including patients 10 and 17—exhibited an increased LVMI, suggestive of early cardiac remodeling.

Overall, overt kidney or cardiac disease was uncommon in this pediatric cohort, but early signs of organ involvement were detectable in a significant subset, highlighting the importance of routine monitoring from a young age (Table 2).

### 3.3. Correlation Analysis of Clinical Indicators

Significant sex-related differences were observed in renal and cardiac parameters. Male patients exhibited markedly lower α-GalA activity compared with females (0.36 ± 0.03 vs. 1.76 ± 0.98 μmol/L/h; t = −3.82; *p* = 0.01), while Lyso-GL-3 levels were substantially elevated in males (73.06 ± 11.40 vs. 3.68 ± 3.96 ng/mL; t = 12.71; *p* < 0.001). The LVMI was significantly higher in males (40.83 ± 6.52 vs. 29.57 ± 7.15 g/m2.7; t = 3.30; *p* = 0.02). By contrast, no significant sex differences were detected for cTnI (0.05 ± 0.03 vs. 0.05 ± 0.05 ng/mL; *p* = 0.91) or eGFR (134.91 ± 36.25 vs. 122.64 ± 28.61 mL/min/1.73 m^2^; *p* = 0.32) (Table 3).

Correlation analysis revealed a strong negative association between α-GalA activity and Lyso-GL-3 levels (r = −0.70; *p* < 0.001). In contrast, no significant correlations were observed between α-GalA and cardiac or renal parameters, including cTnI (r = −0.20; *p* = 0.38), eGFR (r = −0.08; *p* = 0.75), or LVMI (r = −0.42; *p* = 0.06). However, Lyso-GL-3 showed a significant positive correlation with LVMI (r = 0.44; *p* = 0.04), suggesting that higher Lyso-GL-3 concentrations may be associated with increased left-ventricular mass (Table 4).

### 3.4. Exosome Characterization

**Figure 1:**
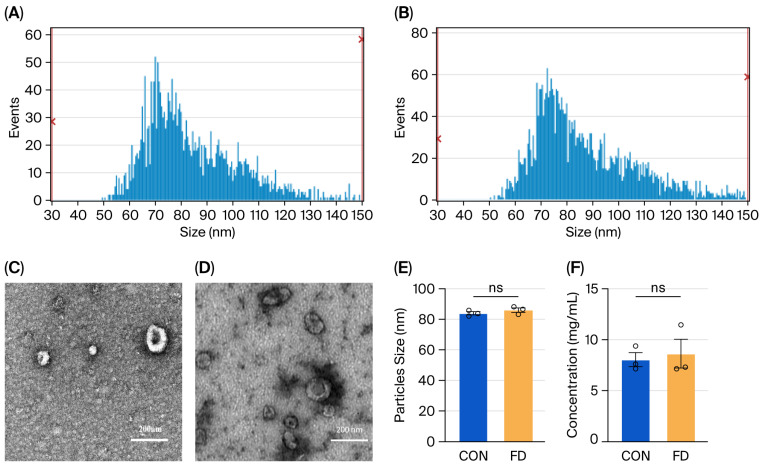
Characterization of plasma-derived exosomes in control and FD groups (**A**,**B**). Size distribution of exosomes from CON and FD plasma. (**C**,**D**) TEM images showing spherical or oval vesicles (30–150 nm) enclosed by a lipid bilayer. Scale bar = 200 nm. (**E**,**F**) Quantitative analysis of exosome size and particle concentration in CON and FD groups. No significant (ns) differences were detected (*p* > 0.05).

Exosomal morphology was assessed using TEM. Plasma-derived exosomes exhibited a uniform spherical or oval shape surrounded by a single lipid bilayer (Figure 1C). Particle diameters ranged from 30 to 150 nm, consistent with reported exosomal dimensions. The mean diameters were 83.80 nm in healthy controls (CON) and 86.56 nm in FD patients, with no significant difference in size distribution between groups (Figure 1A,B,E). Exosomes were further validated by Western blot using TSG101 as a positive marker and calnexin as a negative control (Figure S1).

### 3.5. Protein Analysis of Exosomes from FD and Control Groups

A total of 2553 proteins were identified by searching against a comprehensive protein database. Differential expression between group FD and group CON was evaluated using *t*-tests based on relative quantification values. Proteins with a fold change ≥1.5 and *p* < 0.05 were classified as significantly upregulated (n = 44), while those with a fold change ≤0.67 and *p* < 0.05 were considered downregulated (n = 144). The full dataset with adjusted *p*-values is provided in Supplementary Table S2. 

**Figure 2:**
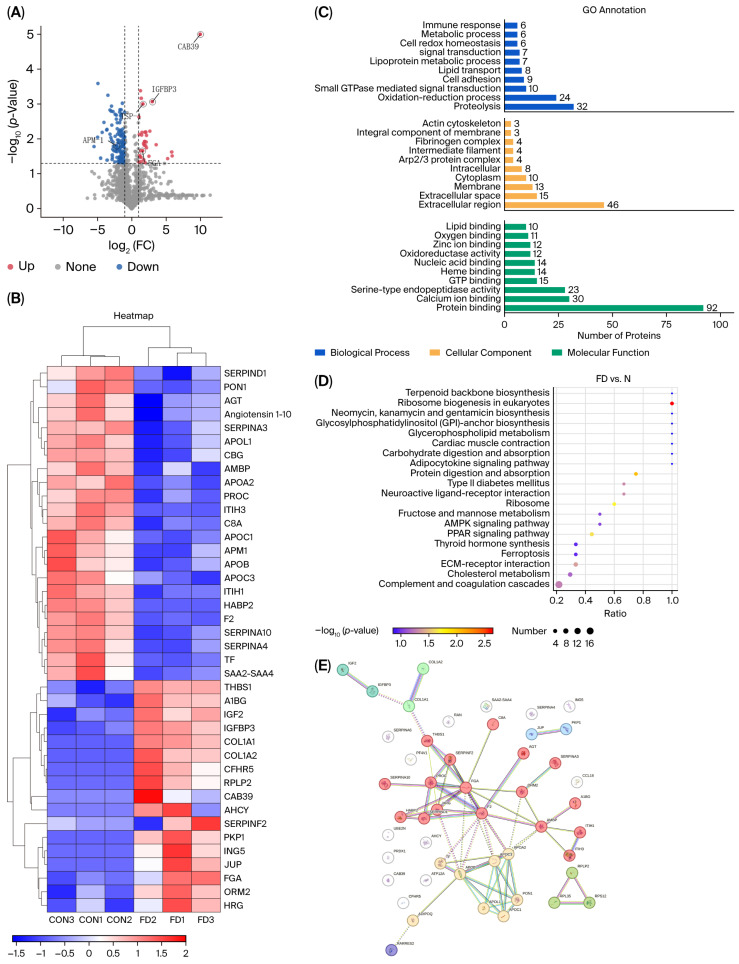
Proteomic profiling of plasma-derived exosomes in FD and CON groups. (**A**) Volcano plot of differentially expressed proteins. (**B**) Heatmap showing sample clustering. (**C**) GO enrichment analysis. (**D**) KEGG pathway enrichment analysis. (**E**) PPI network generated using STRING, the lines in the figure follow the default settings of the STRING database: a greater number of lines indicates higher confidence in the protein–protein interaction.

Exosomal proteomic analysis revealed significant alterations in proteins related to renal pathology. THBS1, implicated in tubulointerstitial remodeling and inflammation, and CFHR5, associated with renal disease progression, were significantly upregulated in the FD group. Conversely, several proteins with renoprotective or disease-related functions were downregulated, including SERPINA10 (a renoprotective protein), A1BG, angiotensin 1–10 (involved in blood pressure regulation via ACE), APOL1 (associated with glomerulosclerosis and nephrotic syndrome), and AMBP (protective against oxidative stress). Proteins associated with lipid metabolism and cardiovascular health were also reduced, including ADIPOQ (also known as APM1, associated with cardiovascular disease) and IGFBP3 (a marker reflective of renal impairment). Other downregulated proteins consisted of APOF, APOA2, APOC1, APOC3, APOB, SAA2-SAA4 (plasma amyloid proteins), and PON1 (Figure 2B). Cluster analysis revealed clear segregation of FD and CON samples, underscoring a disease-specific exosomal proteomic profile and highlighting molecular signatures related to renal injury, inflammation, and metabolic dysregulation.

Gene Ontology (GO) analysis revealed that differentially expressed proteins were associated with extracellular matrix structural constituents, metallopeptidase activity, serine-type peptidase inhibitor activity, zinc-ion binding, and transition-metal-ion binding. Biological processes included organic substance metabolism, nitrogen compound catabolism, and macromolecule metabolism, with localization enriched in the nucleus, ribonucleoprotein complexes, extracellular regions, and processing bodies (Figure 2C).

KEGG pathway analysis revealed significant enrichment in ribosome biogenesis, protein digestion and absorption, PPAR and AMPK signaling, extracellular matrix–receptor interactions, type II diabetes mellitus, lipid metabolism, complement and coagulation cascades, and cardiac muscle contraction (Figure 2D).

Protein–protein interaction (PPI) analysis using the STRING database (http://string-db.org/, accessed on 7 September 2025) led to the construction of an interaction network. Prominent functional modules included apolipoproteins (e.g., APOA2, APOC3, APOB, APOL1, APOC1, PON1, TF, and APM1) and proteins associated with the complement and coagulation systems (such as THBS1, SERPINF2, FGA, F2, AMBP, HRG, C8A, ORM2, and PROC) (Figure 2E).

### 3.6. Validation of Differential Proteins

**Figure 3:**
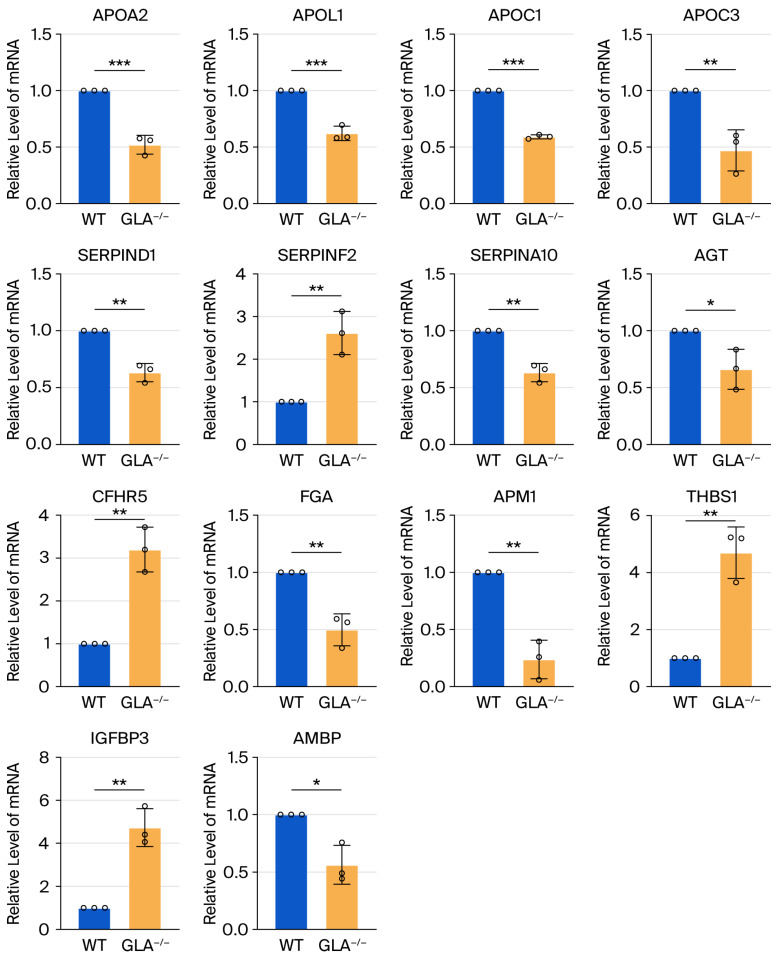
Validation of exosomal proteomic findings in HEK293 cells. Relative mRNA expression of selected genes (*APOA2*, *APOL1*, *APOC1*, *APOC3*, *SERPIND1*, *SERPINF2*, *SERPINA10*, *AGT*, *CFHR5*, *FGA*, *APM1*, *THBS1*, and *AMBP*) in *Gla*^−/−^ HEK 293, quantified by RT-PCR. *, *p* < 0.05; **, *p* < 0.01; ***, *p* < 0.001.

RT-PCR was performed in HEK 293 cells to validate exosomal proteomic findings (n = 4). qPCR targets were derived from exosomal proteomic findings and represent proteins linked to kidney and heart pathology, including upregulation of THBS1 (4.704 ± 0.934 fold vs. WT; *p* < 0.01), IGFBP3 (4.732 ± 0.880; *p* < 0.001), CFHR5 (3.199 ± 0.521; *p* < 0.01), and SRRPINF2 (2.615 ±0.506; *p* < 0.01), and downregulation of APM1 (ADIPOQ 0.238 ± 0.169; *p* < 0.01), APOA2 (0.521 ± 0.083; *p* < 0.001), APOL1 (0.621 ± 0.064; *p* < 0.001), APOC1 (0.591 ± 0.018; *p* < 0.001), APOC3 (0.471 ± 0.182; *p* < 0.01), AMBP (0.5564 ± 0.169; *p* < 0.05), SERPIND1 (0.633 ± 0.081; *p* < 0.01), SERPINA10 (0.632 ± 0.078; *p* < 0.01), AGT (0.661 ± 0.176; *p* < 0.05), and FGA (0.498 ± 0.139; *p* < 0.01) (Figure 3).

**Figure 4:**
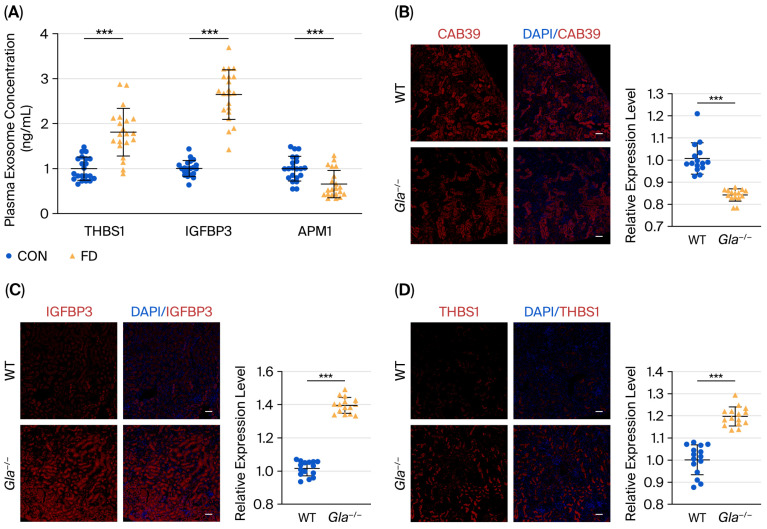
Validation of exosomal protein alterations in plasma and kidney tissues. (**A**) ELISA quantification of plasma exosomes from 21 FD patients showing increased THBS1 and IGFBP3 levels and decreased APM1 levels. (**B**–**D**) Immunofluorescence staining of kidney tissues from *Gla*^−/−^ mice (B6;129-*Gla*^tm1Kul^/J; stock no. 003535, Jackson Laboratory), demonstrating reduced CAB39 expression and elevated IGFBP3 and THBS1 expression. Fluorescence intensity of the target protein was quantified in renal tubule regions per group using image analysis software (n = 5; three fields of vision were counted per mouse). ***, *p* < 0.001.

Further validation in patient-derived plasma exosomes (n = 21) using ELISA revealed significantly increased levels of THBS1 and IGFBP3, whereas APM1 levels were markedly reduced in FD patients compared with controls (Figure 4A). Consistently, immunofluorescence staining of kidney tissues from *Gla*^−/−^ mice demonstrated decreased CAB39 (0.842 ± 0.028; *p* < 0.001) expression along with elevated IGFBP3 (1.396 ± 0.048; *p* < 0.001) and THBS1 (1.197 ± 0.043, *p* < 0.001) expression in renal tubules, supporting the relevance of these exosomal signatures to tissue-level changes in Fabry nephropathy (Figure 4B–D).

## 4. Discussion

Delayed diagnosis remains a major challenge in FD, often resulting in missed opportunities for early intervention and irreversible organ injury. In our cohort, the diagnostic latency ranged from 8 months to 7 years; such delays may reduce the effectiveness of enzyme--replacement therapy (ERT) and worsen clinical outcomes [4,12]. The efficacy of ERT is highly time-dependent; once structural lesions such as glomerulosclerosis, interstitial fibrosis, or myocardial hypertrophy are established, ERT typically slows progression but rarely restores renal function or reverses cardiac remodeling [13].

Analysis of 21 pediatric FD patients revealed that most maintained normal or supranormal glomerular filtration, yet several already showed early evidence of organ involvement, including proteinuria, microalbuminuria, hematuria, an elevated LVMI, or increased cTnI levels. These subclinical changes indicate that conventional markers such as creatinine and eGFR lack sensitivity for detecting early functional decline in children [11,14]. Proteins derived from plasma exosomes can report molecular changes not captured by routine assays and, therefore, constitute promising candidates for early diagnostic biomarkers [15,16].

Neither Lyso-GL-3 nor α-Gal A levels showed a clear correlation with age in our cohort. This finding is consistent with previous studies highlighting genotype, sex, and treatment status as primary determinants. Given this well-established pattern and the limited sample size, we did not perform additional age-based correlation analyses, focusing instead on phenotype- and organ-specific interpretations of the exosomal profiles [17,18].

Exosomal proteomics in this study identified over 2500 proteins. Pathways showing significant perturbation included PPAR and AMPK signaling, lipid metabolism, and complement and coagulation cascades, all of which are central to renal and cardiovascular homeostasis [19] and provide molecular evidence of early renal involvement. In pediatric plasma exosomes, THBS1 and CFHR5 were markedly upregulated [20]. THBS1, enriched in the tubulointerstitial compartment, activates latent TGF-β1 and promotes interstitial fibrosis, supporting its role as a sensitive marker of subclinical renal fibrogenesis [21,22]. CFHR5, a regulator of the alternative complement pathway implicated in complement-mediated nephropathies, suggests complement-related glomerular injury in FD [23,24].

In contrast, several cytoprotective proteins, including SERPINA10, A1BG, and AMBP, were downregulated and exosomal angiotensin I-10 was reduced, indicating impaired antioxidant/anti-inflammatory defenses and dysregulation of the renin–angiotensin system; these changes are consistent with the observation of proteinuria and microalbuminuria despite preserved serum creatinine [25]. In the cardiovascular domain, concurrent upregulation of THBS1 and downregulation of adiponectin (APM1) reflect an imbalance between profibrotic and protective signaling. THBS1 facilitates myocardial fibrosis via TGF-β activation and MMP/myofibroblast pathways, whereas adiponectin signals through AMPK/PPAR-α to exert anti-inflammatory, antifibrotic, and metabolic protective effects. Reduced adiponectin levels correlated with an increased LVMI and subclinical myocardial involvement [22,26,27,28,29].

Collectively, reciprocal changes in exosomal THBS1 and adiponectin, together with complement activation and loss of cytoprotective factors, indicate that profibrotic and inflammatory drivers outweigh protective pathways in early FD. These observations provide mechanistic insights and nominate exosomal THBS1, CFHR5, and adiponectin as candidate biomarkers for risk stratification and therapeutic monitoring; targeted mechanistic validation, including molecular docking and in vitro/in vivo functional studies, is required.

GO enrichment linked differentially expressed exosomal proteins in pediatric FD to extracellular matrix organization, metalloproteinase activity, serine protease inhibition, and metal-ion binding, functions integral to matrix remodeling, enzyme regulation, and metabolic homeostasis, and closely relevant to renal pathology. KEGG analysis highlighted ribosome biogenesis, protein digestion and absorption, PPAR and AMPK signaling, ECM–receptor interactions, type 2 diabetes mellitus, lipid metabolism, complement–coagulation cascades, and cardiac contraction pathways. PPAR and AMPK pathways regulate fatty acid oxidation, energy metabolism, and anti-inflammatory responses. Enrichment of these pathway components in exosomes, together with decreased adiponectin, is compatible with impairment of protective metabolic signaling and a shift toward metabolic stress and profibrotic states [19,30,31]. Interpretation of certain enriched terms (e.g., “type 2 diabetes” and “cardiac contraction”) requires caution, since such annotations may reflect broad enrichment of metabolic proteins rather than disease-specific mechanisms. Pathological inferences drawn from these terms demand targeted experimental validation.

The PPI network analysis identified an apolipoprotein-centered lipid transport module and a coagulation/complement module (FGA, F2, etc.), while THBS1 and IGFBP3 emerged as central nodes linking fibrotic and growth-factor regulatory processes, indicating that FD exosomes capture interrelated mechanisms of fibrosis, inflammation, and vascular dysfunction [32,33,34].

In summary, functional enrichment and network analyses suggest that exosomal proteins reflect systemic disease mechanisms in FD, bridging renal fibrosis, metabolic dysregulation, and cardiovascular remodeling. These molecular signatures not only mirror early clinical manifestations such as proteinuria, reduced eGFR, and left-ventricular hypertrophy, but also hold promise as biomarkers for disease progression and therapeutic monitoring. Future longitudinal studies integrating exosomal proteomics with imaging and genetic screening will be essential to establish their role in the precise diagnosis and personalized treatment of FD. A key limitation is that all samples were from symptomatic, untreated patients; pre-symptomatic cases were not included. As Lyso-Gb3 levels are known to rise before symptom onset, further studies are needed to assess whether exosomal markers exhibit similar early trends. Overall, these findings are exploratory and hypothesis-generating, and prospective validation is required to assess their clinical utility.

## 5. Conclusions

In conclusion, exosomal proteomic analysis in pediatric FD revealed key molecular alterations linked to renal and cardiovascular injury. Among the differentially expressed proteins, THBS1 and CFHR5 emerged as potential markers of renal fibrosis and complement-mediated glomerular injury, while IGFBP3 reflected growth factor dysregulation associated with both renal and cardiac remodeling. In contrast, the downregulation of APM1 highlighted loss of metabolic and cardioprotective signaling, and reduced CAB39 suggested impaired AMPK-mediated stress resistance. Together, these proteins delineate a pathophysiological imbalance between profibrotic drivers and protective mechanisms in FD. From a clinical perspective, such exosomal biomarkers hold promise for improving early diagnosis, monitoring subclinical organ involvement, and guiding therapeutic timing, particularly for interventions such as ERT. Their integration into clinical practice may enable earlier risk stratification, closer surveillance of renal and cardiac complications, and more personalized management strategies to optimize long-term outcomes in FD.

## Figures and Tables

**Table 1:** Demographic, genetic, biochemical, and clinical characteristics of pediatric FD patients.

	Diagnosis Age	Diagnosis Years (Years)	Gender	*GLA* Gene Mutation	Exon	α-Gal A Mutation	Mutation Origin	ACMG Classification *	α-Gal A Levels ^☆^	Lyso-GL-3 Levels ^▲^	Acroparesthesia	Hypohidrosis or Anhidrosis	Skin Vascular Keratomas	Gastrointestinal Symptoms	Screening Method
1	14Y	3	Male	c.424T>C	E3	p.Cys142Arg	Mother	Pathogenic	0.32	80.45	Yes	Yes	No	Yes	High-risk screening
2	16Y9M	-	Female	c.335G>A	E2	p.vArg112His	Father	Pathogenic	2.1	0.55	No	No	No	No	Familial screening
3	6Y	-	Female	c.335G>A	E2	p.Arg112His	Father	Pathogenic	1.31	0.51	No	No	No	No	Familial screening
4	11Y10M	6	Male	c.644A>G	E5	p.Asn215Ser	Mother	Pathogenic	0.6	7.01	No	Yes	No	No	High-risk screening
5	11Y5M	1	Male	c.140G>A	E1	p.Trp47Ter	Mother	Likely pathogenic	0.4	92.98	Yes	Yes	Yes	No	High-risk screening
6	13Y6M	1	Male	c.3G>A	E1	p.Met1?	Spontaneous mutation	Likely pathogenic	0.34	74.02	Yes	Yes	No	No	High-risk screening
7	9Y9M	1	Male	c.100A>G	E1	p.Asn34His	Mother	Pathogenic	0.33	64.05	Yes	Yes	No	No	Familial screening
8	9Y9M	0.7	Male	c.100A>G	E1	p.Asn34His	Mother	Pathogenic	0.4	65.03	Yes	Yes	No	No	Familial screening
9	11Y11M	-	Female	c.100A>G	E1	p.Asn34His	Mother	Pathogenic	1.64	2.77	No	No	No	No	Familial screening
10	8Y1M	1	Male	c.776C>T	E5	p.Pro259Leu	Mother	VUS	0.35	75.16	Yes	Yes	No	No	Familial screening
11	12Y9M	5	Male	c.334C>T	E2	p.Arg112Cys	Mother	Pathogenic	0.35	59.73	Yes	Yes	Yes	No	High-risk screening
12	10Y10M	1.5	Male	c.334C>T	E2	p.Arg112Cys	Mother	Pathogenic	0.45	69.44	Yes	Yes	No	Yes	Familial screening
13	8Y2M	0.7	Male	c.782G>T	E5	p.Gly261Val	Mother	Pathogenic	0.28	71.33	Yes	Yes	No	Yes	High-risk screening
14	7Y11M	-	Female	c.782G>T	E5	p.Gly261Val	Mother	Pathogenic	1.6	4.52	No	Yes	No	No	Familial screening
15	12Y9M	4.1	Male	c.72G>A	E1	p.Trp24Ter	Mother	Pathogenic	0.32	115.63	Yes	Yes	No	Yes	High-risk screening
16	9Y8M	1	Female	c.640–801G>A	I4	-	Father	Pathogenic	3.7	1.69	Yes	No	No	Yes	High-risk screening
17	13Y9M	2	Male	C.486 G>A	E3	p.Trp162 Ter	Mother	Pathogenic	0.67	72.6	Yes	Yes	No	Yes	Familial screening
18	17Y	7	Male	c.1072_1074delGAG	E7	p.Glu358del	Mother	Pathogenic	0.26	65.57	Yes	Yes	Yes	Yes	High-risk screening
19	8Y6M	1	Male	c.454_456dupTAC	E3	p.Tyr152dup	Mother	Pathogenic	0.86	66.96	Yes	Yes	Yes	No	Familial screening
20	10Y10M	-	Female	c.1197G>A	E1	p.Trp399Ter	Father	Pathogenic	1.49	3.73	Yes	No	No	No	Familial screening
21	8Y6M	-	Female	c.187T>C	E1	p.Cys63Arg	Father	Likely pathogenic	0.49	11.96	No	No	No	No	Familial screening

Abbreviations: *, ACMG Classification: Pathogenicity of genetic variants was evaluated according to the American College of Medical Genetics and Genomics (ACMG) guidelines, including five categories: pathogenic, likely pathogenic, uncertain significance (VUS), likely benign, and benign; ☆, normal reference range for α-Gal A activity: 2.40–17.65 μmol/L/h; ▲, normal reference range for Lyso-GL-3: <1.11 ng/mL; ?, denotes an uncertain or predicted effect.

**Table 2:** Renal and cardiac function in pediatric patients with FD.

	Scr (μmol/L)	eGFR (mL/min/1.73 m^2^)	UACR (mg/g·Cr)	RBC/HPF	24 h UP (mg)	cTnI (ng/mL)	LVMI (g/m^2.7^)
1	85	74.7	26.7	>200	209	0.09	44.1
2	73	82.0	10.3	6	81.2	0.01	39.4
3	51	88.7	8.7	4	46.3	0.01	40.4
4	51	114.5	9.6	6	159.9	0.08	42.3
5	35	152.3	22.2	6	81.8	0.05	42
6	31	175.4	11.4	5	54.1	0.06	32.2
7	37	127.3	10.6	2	81.6	0.02	39.5
8	46	123.8	9.3	2	36.2	0.01	34
9	38	154.6	8.3	0	41.8	0.04	26.5
10	30	176.4	30.3	7	101.8	0.05	51.7
11	46	119.0	7.7	4	91.9	0.15	39.6
12	34	141.7	7.9	4	80.3	0.03	51
13	34	138.4	8.21	4	65.2	0.04	43.3
14	38	135.4	9.0	0	74.5	0.01	26.9
15	56	102.3	3.9	0	83.4	0.07	30.8
16	38	125.8	2.7	0	61.1	0.08	24.7
17	54	105.4	2.2	0	106.1	0.02	52.3
18	64	103.2	19.0	0	86.7	0.19	26.9
19	38	142.2	6.0	0	104.8	0.01	40.1
20	38	152.7	5.4	0	79.6	0.06	25.5
21	41	119.3	10.6	0	144.1	0.13	23.6

Abbreviations: eGFR, estimated glomerular filtration rate; LVMI, left-ventricular mass index; cTnI, cardiac troponin I. Reference ranges: cTnI ≤ 0.4 ng/mL; eGFR ≥ 90 mL/min/1.73 m^2^; urine albumin-to-creatinine ratio: 0–30 mg/g; 24 h urinary protein < 150 mg; LVMI < 38 g/m^2.7^.

**Table 3:** Sex-based differences in biochemical and clinical parameters among pediatric FD patients.

Variable	Group	Mean ± SD	Mean Difference (95% CI)	t	*p*
α-GalA (μmol/L/h)	Male	0.36 ± 0.03	−1.41 (−2.31 to −0.51)	−3.82	0.01 **
	Female	1.76 ± 0.98			
Lyso-GL-3 (ng/mL)	Male	73.06 ± 11.40	69.38 (56.03 to 82.74)	12.71	0.00 **
	Female	3.68 ± 3.96			
cTnI (ng/mL)	Male	0.05 ± 0.03	0.00 (−0.06 to −0.06)	0.11	0.91
	Female	0.05 ± 0.05			
eGFR (mL/min/1.73 m^2^)	Male	134.91 ± 36.25	12.27 (−15.39 to −39.93)	1.09	0.32
	Female	122.64 ± 28.61			
LVMI (g/m^2.7^)	Male	40.83 ± 6.52	11.26 (2.92 to 19.60)	3.30	0.02 *
	Female	29.57 ± 7.15			

* *p* < 0.05; ** *p* < 0.01.

**Table 4:** Correlation analysis between α-Gal A activity, Lyso-GL-3, and organ function parameters in pediatric FD patients.

Variable	α-GalA		Lyso-GL-3	
	r	*p*	r	*p*
α-GalA (μmoL/L/h)	-	-	−0.70	0.00 **
Lyso-GL-3 (ng/mL)	−0.70	0.00 **	-	-
cTnI (ng/mL)	−0.20	0.38	0.09	0.70
eGFR (mL/min/1.73 m^2^)	−0.08	0.75	0.11	0.65
LVMI (g/m^2.7^)	−0.42	0.06	0.44 *	0.04 *

Correlation coefficients (r) were calculated using Pearson’s test. * *p* < 0.05; ** *p* < 0.01.

## Data Availability

Raw proteomics data have been deposited in PRIDE (accession number: [PXD069403]).

## Supplementary Materials

The following supporting information can be downloaded at: https://www.mdpi.com/article/10.3390/biomedicines13112598/s1, Table S1. The primers used in the study; Table S2. Full dataset with adjusted *p*-values; Figure S1. Western blot analysis of exosomal marker in human plasma-derived exosomes; Figure S2. Validation of CRISPR-Cas9–mediated gene editing in HEK293 cells.

## Abbreviations

The following abbreviations are used in this manuscript:

FD	Fabry disease
α-Gal A	α-galactosidase A
GL-3	globotriaosylceramide
Lyso-GL-3	glycosphingolipids
