# Supplementary material for: Exosome-Based Proteomic Profiling for Biomarker Discovery in Pediatric Fabry Disease: Insights into Early Diagnosis Monitoring
Source: Biomedicines. 2025 Oct 23;13(11):2598. doi: 10.3390/biomedicines13112598 (PMC12650516; doi:10.3390/biomedicines13112598)
Supplement: Supplementary file 1 [file biomedicines-13-02598-s001.zip › Supplementary figures.pptx]

## Slide 1
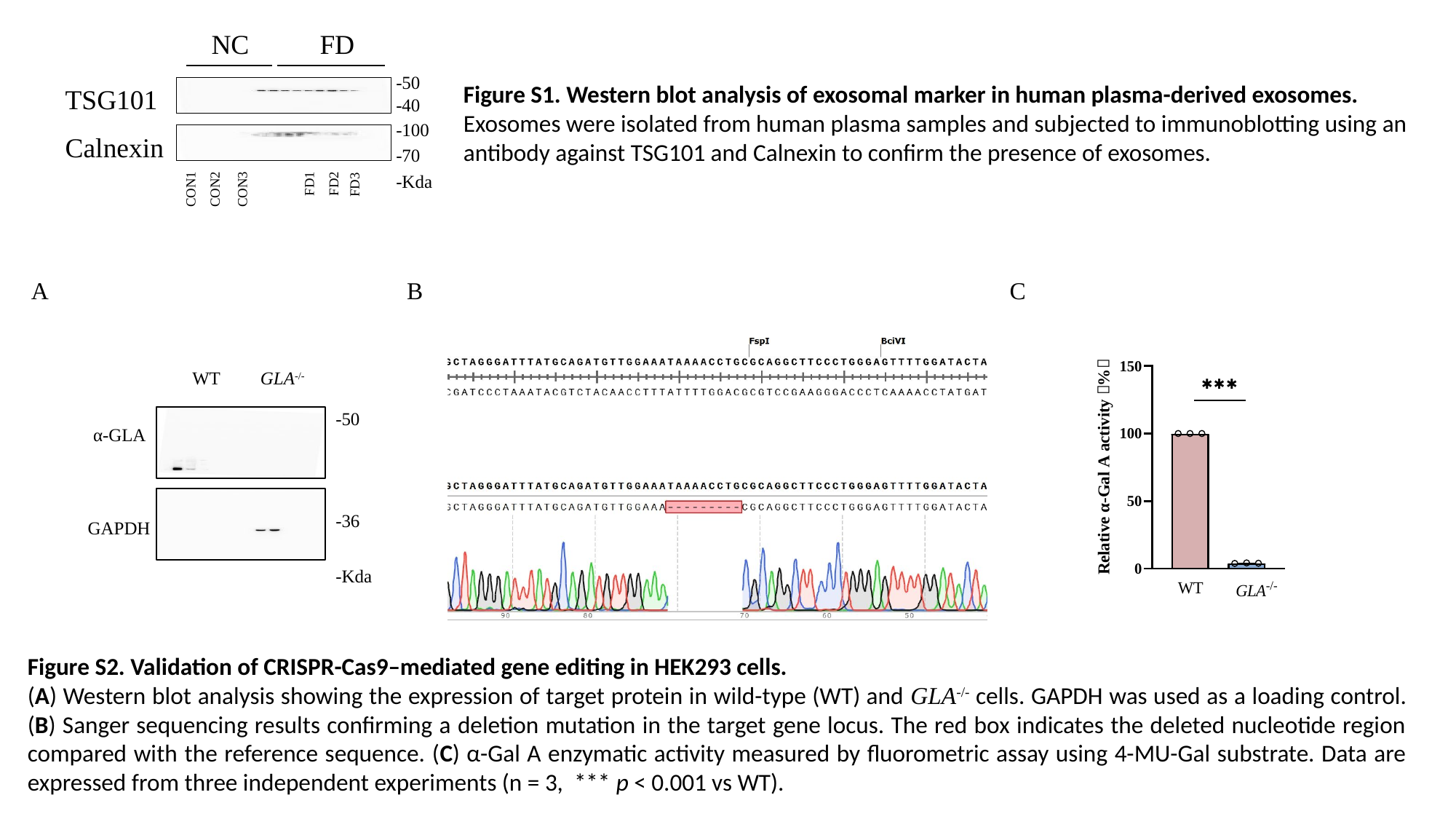

NC
FD
-50
TSG101
-40
-100
Calnexin
-70
-Kda
CON1
CON2
CON3
FD1
FD2
FD3
Figure S1. Western blot analysis of exosomal marker in human plasma-derived exosomes.
Exosomes were isolated from human plasma samples and subjected to immunoblotting using an antibody against TSG101 and Calnexin to confirm the presence of exosomes.
A
B
C
WT
GLA-/-
-50
α-GLA
-36
GAPDH
-Kda
Figure S2. Validation of CRISPR-Cas9–mediated gene editing in HEK293 cells.
(A) Western blot analysis showing the expression of target protein in wild-type (WT) and GLA-/- cells. GAPDH was used as a loading control. (B) Sanger sequencing results confirming a deletion mutation in the target gene locus. The red box indicates the deleted nucleotide region compared with the reference sequence. (C) α-Gal A enzymatic activity measured by fluorometric assay using 4-MU-Gal substrate. Data are expressed from three independent experiments (n = 3, *** p < 0.001 vs WT).
