# Supplementary material for: Exosome-Based Proteomic Profiling for Biomarker Discovery in Pediatric Fabry Disease: Insights into Early Diagnosis Monitoring
Source: Biomedicines. 2025 Oct 23;13(11):2598. doi: 10.3390/biomedicines13112598 (PMC12650516; doi:10.3390/biomedicines13112598)
Supplement: Supplementary file 1 [file biomedicines-13-02598-s001.zip › Supplementary Table S1.pdf]

Supplement Table S1. The primers used in the study

Gene Name	Forward Primer (5′→3′)	Reverse Primer (5′→3′)
APOA2	CTGTGCTACTCCTCACCATCT	CTCTCCACACATGGCTCCTTT
APOC1	TCCAGTGCCTTGGATAAGCTG	GGCTGATGAGTTCCCGAGC
APOC3	CCGCCAAGGATGCACTGAG	CCGCCAAGGATGCACTGAG
APOL1	GAGGTGAGGGAGTTTTTGGGT	TCGTGTGAGTTGGTAAGTATTGC
FGA	TGTCGAGGGTCATGCAGTAG	CAAGTTGCTTCTGCTGATCTTCA
SERPIND1	GGACGACGACTATCTGGACCT	CATCAGAGTCTGTCGGGGAAA
SERPINF2	GTGCCCGTGGAAATGATGC	AAAGTGGGTGGGTACAAGGAC
SERPINA10	TGAGGCACGATGGCAACAT	GAGCCCTCTCTTGATCTGGG
THBS1	TGCTATCACAACGGAGTTCAGT	GCAGGACACCTTTTTGCAGATG
APM1	GGCTTTCCGGGAATCCAAGG	TGGGGATAGTAACGTAAGTCTCC
CFHR5	AGAAAGCTACAAAGTTGGAGACG	TGACCACCCAAATTGGTAACATT
AMBP	CTCTCGGATCTATGGGAAGTGG	CGTGCTCACTGTCATCCTGTC
AGT	CTCCAATTCAGGCCAAGACAT	TGTCAAGTTTTGCAGCGACTA
B-ACTIN	CACCATTGGCAATGAGCGGTTC	AGGTCTTTGCGGATGTCCACGT
